# National-Level Biosafety Norms Needed for Dual-Use Research

**DOI:** 10.3389/fpubh.2014.00084

**Published:** 2014-07-14

**Authors:** Gigi Kwik Gronvall

**Affiliations:** ^1^UPMC Center for Health Security, Baltimore, MD, USA

**Keywords:** DURC, dual-use research, gain-of-function, biosafety, biological research norms

Dual-use concerns – that legitimate research has the potential to be misused – are inherent in life sciences research. In the past 15 years, numerous scientific papers have raised security questions, and split opinions of scientists, ethicists, and policymakers on whether the research should have been performed or published. Some of the most high-profile examples include the addition of an immunomodulatory gene into the mousepox virus genome, which made the mousepox vaccine ineffective (and suggested that similar manipulations to the smallpox virus genome could make the smallpox vaccine ineffective); the synthesis of poliovirus, the 1918 influenza strain, and the synthesis of a bacterial cell; and “gain of function” (GOF) work on influenza viruses to explore whether H5N1 and other strains have the potential to become transmissible in humans (1–6). Given the increasing ease of manipulating and synthesizing genetic material, and the continued expansion of biological research globally, additional dual-use concerns are certain to arise in the future.

In response to these challenges, policies have been developed in the US to allow a thoughtful pause before beginning or publishing specific areas of research, to consider which aspects of the research are potentially problematic, and evaluate what can be done to mitigate concerns. (7, 8) Though such policies may become more common in biological research, it will be difficult to create hard-and-fast rules about whether or not to conduct potentially dual-use research and publish it in the open scientific literature, because what to do about the work is inextricably tied to the specifics of the research in question. Decisions of whether to fund, perform research on, or publish the next dual-use research of concern article, whether it involves influenza, a different pathogen, or something that is not a pathogen at all, will likely be tipped one way or another by a mix of qualities that are difficult to predict. Some of these qualities are the technical specifics of the research in question; the researchers involved; the urgency of the public health threat that the research is trying to address; an assessment of the danger that the information could be applied toward a biological weapon; and an assessment of the soundness or importance of the research. It is unlikely that in considering these factors, consensus will emerge about what the right course of action should be, or agreement about whether the work will yield important scientific or public health advances. The lack of consensus may lead to some types of work not being funded by one government but pursued by another, or journals with different standards for publication.

Some dual-use research raises concerns, however that can be more easily and broadly addressed than the potential for misuse: the potential for accident. The laboratories, which first demonstrated that H5N1 avian influenza has the potential to become transmissible in mammals have high levels of biosafety training, top-of-the-line equipment, engineered controls, and health monitoring of the researchers performing the work. Yet as GOF influenza research is repeated elsewhere, or even becomes commonplace, how can people be assured that the same level of attention will be paid? Biosafety is particularly important in these cases because of the potential of an accident to spark a pandemic. Most accidents in biological laboratories are likely to be limited to the researchers involved and possibly their close contacts, but laboratory acquired infections with transmissible pathogens, such as non-circulating human influenza strains, SARS, or engineered influenza strains could have consequences that go well beyond the laboratory (9, 10).

The good news is that safety is more objectively measured than dual-use research, and there are practical systems to put into place that could raise confidence that concerns are being addressed. There is excellent guidance available for individual researchers, laboratories, and research institutions to adhere to high biosafety practices, as well as biosafety professional training. There are international standards for BSL-1, BSL-2, BSL-3, and BSL-4 labs including what engineering controls should be in place in each level of biocontainment, as well as to manage biorisks within a research institution. (11, 12) The World Health Organization, the Centers for Disease Control and Prevention, professional organizations, and other institutions aim to bring technical information to practitioners, enhance laboratory safety practice, and promote biosafety standards (13–17).

Yet while technical guidance for researchers and institutions is in abundance, a key piece is missing: *national-level* norms for the safety systems necessary to perform such consequential research, to make biosafety a political priority. The next time there is concern about GOF or some other potentially concerning research, it would be helpful to know that the research took place in an environment where there are national standards for the work, including for equipment maintenance, worker safety training, health monitoring, surveillance, and other myriad activities to help keep the researchers and the larger public safe, and that the nation has an adequate surveillance system in place to identify and limit potential outbreaks that could result from such accidents. Without national-level standards for biosafety and interest in making sure that research institutions that perform potentially high-consequence research adhere to those standards, there will remain insufficient incentives to commit the resources required to achieve high levels of biosafety in individual laboratories and institutions.

The problem of setting biosafety standards for GOF work and other, dual-use research with the potential for consequential accidents does not address the dual-use dilemma in the life sciences, in that such research may lower barriers toward making a biological weapon. But, for legitimate scientific research, increasing international accountability for safety could raise barriers to accidentally achieving the same, horrible result. Even the most dangerous pathogen cannot cause harm to populations if it does not escape containment. Nations which fund this type of scientific research should therefore have the systems in place to provide appropriate levels of safety.

## Conflict of Interest Statement

The author declares that the research was conducted in the absence of any commercial or financial relationships that could be construed as a potential conflict of interest.
